# Clinical trial site identification practices and the use of electronic health records in feasibility evaluations: An interview study in the Nordic countries

**DOI:** 10.1177/17407745211038512

**Published:** 2021-08-25

**Authors:** Niina Laaksonen, Mia Bengtström, Anna Axelin, Juuso Blomster, Mika Scheinin, Risto Huupponen

**Affiliations:** 1 Institute of Biomedicine, University of Turku, Turku, Finland; 2 Pharma Industry Finland, Helsinki, Finland; 3 Pharmaceutical Sciences Laboratory, Åbo Akademi University, Turku, Finland; 4 Department of Nursing Science, University of Turku, Turku, Finland; 5 Department of Cardiology, University of Turku and Turku University Hospital, Turku, Finland; 6 Clinical Pharmacology Unit, Turku University Hospital, Turku, Finland; 7 Clinical Research Services Turku – CRST Oy, Turku, Finland

**Keywords:** Site identification, trial sponsor, electronic health record, electronic health records, patient count estimates, feasibility evaluation, Nordic countries

## Abstract

**Introduction:**

Feasibility evaluations are performed to create the best possible starting point for the set-up and execution of a clinical trial, and to identify any obstacles for successful trial conduct. New digital technologies can provide various types of data for use in feasibility evaluations. There is a need to identify and compare such data sources for trial site identification and for evaluating the sites’ patient recruitment potential. Especially, information is needed on the use of electronic health records. We investigated how different data sources are used by pharmaceutical companies operating in the Nordic countries for identifying trial sites and for evaluating their potential to recruit trial participants.

**Methods:**

This was a semi-structured qualitative interview study with 21 participants from pharmaceutical companies and contract research organizations operating in Finland, Sweden, Denmark and Norway. Qualitative content analysis was applied.

**Results:**

For identifying countries and trial sites on a global level, the trial sponsors mostly used databases on previous trial performance. The use of electronic health record data was very limited. Sites’ and investigators’ visibility in various databases was seen as fundamental for their countries becoming selected into new clinical trials. For estimating the sites’ recruitment projections, most sites were seen to base their patient count estimates solely on their previous experience. Some sites had reviewed their electronic health record data, which was considered to increase the accuracy of their recruitment estimates and these sites’ attractivity. Along with dialogs with investigators, the sponsors used various data sources to validate the investigators’ estimates. Legislative obstacles were seen to hinder the use of electronic health record queries for estimation of patient counts.

**Conclusion:**

Visibility in the databases used by trial sponsors is crucial for the countries and sites to be identified. Site selection appears to be based on trust and relationships built from experience, but electronic data provide the support upon which the trust is based. Estimation of the number of potential trial participants is a complex and time-consuming process for both investigators and sponsors. Sponsors seem to favour sites who could support their patient count estimates with electronic health record data as they were quicker in providing the estimates and more reliable than sites with no electronic health record evidence. The patient count evaluation process could be simplified, accelerated and made more reliable with more systematic use of electronic health record evidence in the feasibility evaluation phase. This would increase the accuracy of the patient count estimates and, on its part, contribute to improved recruitment success.

## Introduction

Poor recruitment has been reported to be the most important reason for clinical trial delays,^1^ ultimately also delaying access to new treatments and increasing drug development costs.^2^ In earlier reports, only 31% of the evaluated clinical trials met their original recruitment targets on time^3^ and 19% of the initiated sites were found to be zero-recruiters.^4^ Therefore, more effort should be placed on proper conduct of feasibility evaluations and trial planning, as many barriers in the recruitment of trial participants can be identified prior to trial initiation.^5^

Trial site identification and site selection are important parts of the overall feasibility evaluation. Site qualities examined include availability of trial participants, timely patient recruitment, resources of the site, and site personnel’s interest and commitment.^6^ Site’s access to patients, that is, the capability to identify and reach potential trial participants, is a key reason for failure or success in recruitment.^7^ We investigate here how access to patients is evaluated during feasibility evaluations carried out by trial sponsors in the pharmaceutical industry, how electronic health records (EHRs) are used in this process, and in case they are not used, what are the reasons.

The secondary use of EHR data is regarded as a valuable means to increase the accuracy of recruitment projections.^8,9^ The Nordic countries are technologically advanced in the secondary use of EHR data,^10^ but there is a lack of information on the use of EHR data in both site identification and estimation of availability of potential trial participants.

We conducted a qualitative interview study among representatives of pharmaceutical companies and clinical contract research organizations (hereinafter trial sponsors) operating in the Nordic countries. This study was part of a larger project investigating the use of EHR systems in clinical trials in the Nordic countries. Our focus was on site identification processes of trial sponsors and how they assessed the ability of trial sites to recruit patients, especially the role of EHR in their assessments.

## Methods

### Study design

This was a qualitative descriptive interview study^11^ based on semi-structured interviews^12^ of selected informants, carried out in March to July of 2019. The study methods and results are reported according to the consolidated criteria for reporting qualitative research (COREQ) checklist.^13^

### Participants

The national Pharma Industry associations Pharma Industry Finland, Läkemedelsindustriföreningen (Sweden) and Legemiddelindustrien (Norway) suggested possible interview participants, who were then contacted by email. Danish participants were recruited through personal industry contacts by M.B. and N.L. Participants were eligible if they were working for a pharmaceutical company or a clinical contract research organization and were involved as sponsor representatives in conducting phase I–III clinical trials on pharmaceuticals. The participants were to have an impact on the site identification and patient recruitment process in their company, which was confirmed before the interviews. Participants only involved in phase I trials with healthy volunteers were excluded. Purposive sampling was applied to ensure that professionals across all of four countries were included. Participants were included consecutively until no new meanings were observed from the interviews.^14^

Twenty-eight interviewee candidates were contacted; one refused to participate, three candidates did not respond to email requests and another three did not fulfil the inclusion criteria. Of the 21 interview participants, seven were from Finland, five from Sweden, five from Denmark and four from Norway. As background information, the participants’ titles, experience in clinical trials, time of employment in their current company and contribution to site identification and evaluation processes were collected. Almost all participants (19 out of 21) had operated in the field of clinical trials for more than 10 years. They represented senior-level employees in 17 different companies. Most (71%) had served their current employer for more than 5 years (Table 1).

**Table 1.** Participant characteristics, n = 21.

Position in the company	
Clinical study management	6
Clinical operations	6
Clinical site management	4
Feasibility and recruitment positions	5
Served current employer	
0–5 years	6
6–10 years	7
11–20 years	6
>20 years	2
History with clinical trials	
<10 years	2
11–20 years	9
>20 years	10

### Interview guide and data collection

The participants were asked to select their two most important pre-market clinical drug trials where they had been involved between 2015 and 2018, and in which at least one Nordic country was included. Seven trials did not meet the above criteria (e.g. the trial had not yet started) and one participant had only chosen one trial. Therefore, 34 trials were available for discussion in the interviews.

A qualitative interview guide (Supplementary Material 1) was sent in advance to the participants. In the interviews, the participants were asked to judge whether recruitment on the Nordic level had been successful or unsuccessful (for trials with still ongoing recruitment: on schedule or delayed). If the recruitment period had been prolonged, the recruitment was classified as unsuccessful. Of all 34 trials discussed, 17 recruited successfully and 17 trials were judged as failed by the participants. Trials were mostly in oncology (24%), neurology (18%) and endocrinology (18%), and most were phase III (65%).

The interview guide was tested with one pilot interview, which was included in the analysis, as no major modifications were needed. N.L. performed all interviews and audiorecorded the discussions with the permission of the participants.

### Data handling and analysis

The interviews were transcribed verbatim and managed with NVivo software, version 12 plus (QSR International Inc., USA). The trial profiles and the responses to the categorical questions were collected using REDCap data management software, version 9.1.12.^15^

Inductive qualitative content analysis was applied to the interview data.^16^ The transcripts were read multiple times to obtain an overall impression of their contents. Only the manifest content (the items actually uttered by the participants) of the interviews was analysed. N.L. coded the meaning units based on the research questions and abstracted the codes sharing the same content area into sub-categories and further grouped them to categories and main categories. An agreement on how to sort the codes was developed together with A.A. by a process of reflection and discussion. Examples of quotations and their coding and categorization are provided in Supplementary Material 2.

## Results

Four main categories were formed as described in the following sections.

### Changing landscape of feasibility evaluations

As viewed by the participants, changes in the landscape of clinical trials, for example, the increased need to find certain types of patients with specific mutations, laboratory values, or rare diseases more precisely, have challenged the feasibility evaluation process and the data needed in evaluations. Also the increased use of various types of electronically available data has changed the evaluation process. Two participants out of 21 also noted the need to critically evaluate all available information: if data would contain major errors or omissions, incorrect assumptions might follow.

### Site identification in two layers

We recognized differing site identification practices on global and local levels. Overall, it seemed that site identification is based on information on previous performance of the site, not on defining where the suitable patients are. On the global level, various databases, either the companies’ own or commercial databases such as DrugDev (IQVIA, USA), Citeline (Informa Plc., London, UK), Global Data (GlobalData Plc., London, UK) or public repositories (e.g. National Library of Medicine Clinical Trials Registry, www.clinicaltrials.gov) were seen to have a major role in identifying potential countries and sites. Only a few participants mentioned that, on the global level, prospective countries were also identified by employing commercial EHR technology platforms based on EHR data from healthcare providers (such as TriNetX, TriNetX LLC, USA).

The participants perceived that the Nordic countries lack visibility in the databases on the global level because of their small populations and low trial conduct volumes. The participants also felt that the Nordic countries were not sufficiently marketing their capabilities to the global decision-makers of pharmaceutical companies. The local Nordic subsidiaries were seen to play a key role in such marketing efforts.

According to many interview participants, on the local level, investigator databases were seldom used in the Nordic countries for site identification, and EHR tools were not applied at all. The Nordic countries have limited numbers of investigators, and most of them are already known to the local subsidiaries. Instead, local intelligence, for example, understanding local practices, treatment paths and healthcare systems, was perceived as valuable in site identification. Such local knowledge was considered impossible to capture from any database, but was perceived as being based on the experience of the local country representatives. In other words, they ‘knew’ their countries.

In two-thirds of the trials covered by this study (23 out of 34 trials, Table 2), the method for identifying sites was based on previous collaboration, often supported by other identification methods. Previous collaboration between the trial sponsor and the trial sites did not as such guarantee recruitment success: almost half of the trials covered here (15/34) solely used sites with previous experience, but one-third (5/15) of them still failed in their recruitment. Based on the data, it seems that the site selection methods are not explicitly related to recruitment success or failure (Table 2). However, successfully recruiting trials more often used multiple recruitment methods than trials with failed recruitment, and trials relying on previous collaboration more often succeeded in recruitment than failed. Searches from investigator networks or databases, Internet searches and reviews of publication databases played only minor roles in the site identification process on the local level. Instead of using investigator databases for site identification, some participants used them for evaluating the validity of the patient number estimates provided by the investigators (see section ‘Investigator databases and previous performance data’).

**Table 2.** Methods for identifying trial sites on the Nordic level (n = 34 trials). A single trial could use multiple methods.

	Number of trials	Recruitment successful	Recruitment failed
Existing contacts with sites and investigators + possibly other supporting identification methods	23	14	9
Only existing contacts with sites and investigators	15	10	5
Sites suggested by Key Opinion Leaders, National CoordinatingInvestigators, Principal Investigators or other stakeholders	7	5	2
Recommendation from within the company	4	1	3
Sites known to treat certain types of patients but no previous collaboration	3	0	3
Public database (such as www.clinicaltrials.gov)	3	2	1
Internet search (such as Google)	2	1	1
Investigator network	1	0	1
Publication database review	1	1	0
Commercial investigator database	1	1	0
Not known	3	0	3

### Evaluation of sites’ access to patients

The access to patients, that is, the sites’ capabilities in finding trial subjects, was found to be a process evaluated by both the investigators and the sponsors during the feasibility evaluation.

#### Investigators evaluating the number of potential trial subjects

Most investigators were perceived not to have enough time, interest or information for proper feasibility evaluations; thus, their estimates on potential patient counts often failed quite significantly. According to most participants, investigators usually did not employ EHR data or statistics from previous trials to support their assumptions. The sites using EHR data were considered attractive by the sponsors: they could promptly justify their estimates of potential trial subjects, which together with their earlier recruitment performance offered them a clear advantage. In fact, those sites seemed to be regarded as more reliable in their patient count estimates even if information whether their estimates actually were more accurate than the estimates of those not using EHR data was absent.

The participants also presented some examples of sites that used their EHR data in patient count estimations. For a feasibility evaluation, these sites pre-screened their potential trial subjects in the hospital’s EHR system. Performing this already in the feasibility evaluation phase was perceived as beneficial for both the site and the trial sponsor: the sponsors received more reliable information on the sites’ recruitment capabilities, and the sites saved time at the launch of the trial as they already had the patients pre-screened, which expedited the start of recruitment.

#### Sponsors evaluating the number of potential trial subjects

It became very clear in the interviews that sponsors did not usually accept the investigators’ estimates as such, but tried to evaluate their validity with dialogs and data, as presented in the following sections.

##### Dialogs with the investigator

The participants highlighted that the feasibility evaluation should always be bi-directional: providing proper information to the investigators and listening to their feedback and justification on trial feasibility. Many participants had not used any actual evidence for validating an investigator’s estimations on potential trial subjects; they had only carried out a dialog with the investigator to get a rationale for the investigator-estimated patient count. Some participants admitted that they had not performed thorough enough evaluations on how the investigators had ended up with certain numbers of predicted trial subjects.

##### Requesting site’s EHR evidence

A few participants asked the sites to justify their estimates by showing that they have made a search in the EHR. Several reasons why sites did not use EHR data emerged. Patient count estimates could require monetary compensation paid by the investigator to hospital’s information technology department. Some hospitals required internal approval before the data search, which would have resulted in unacceptable delays as the time frame to reply to feasibility questionnaires is rather short. Overall, to get patient count estimates beyond the investigator’s own patients required additional time and effort from the investigator, without any monetary compensation for this work at time when there was no guarantee that the investigator would be selected to participate in the trial. The use of EHR data for reviewing the availability of potential trial subjects was not only a choice to make or not to make by the investigators. As viewed by the participants, there are legislative restrictions, for example, in the access to and in the secondary use of patient data in the Nordic countries which regulate how investigators can utilize EHR systems for this purpose.

The participants highlighted that the contribution of EHR data in feasibility evaluations is indication-dependent. In trials on chronic diseases, EHR may give information on actual patients potentially identified as suitable for the trial, whereas in acute diseases, for example, stroke, EHR data could be used to reveal how many such patients have been seen by the site in the recent past and hence to estimate the number of potential trial subjects in the near future.

##### Investigator databases and previous performance data

Using previous site performance data, the participants were seeking confidence in the investigator-estimated patient counts. It was perceived as a quick and objective way to validate investigators’ estimates. However, it was possible that data were not comparable with the requirements of a new trial, or did not contain enrolment numbers of the site under evaluation, which complicated the validation.

##### Use of EHR query tools

It became very clear that the sponsors did not commonly use EHR query tools for evaluating potential patient counts in the Nordic countries. The main reason was seen in the legislation restricting access to patient data for such use. For aggregate EHR data (only patient counts), some participants mentioned that hospital management’s interpretation of the legislation and prevailing attitudes as the biggest obstacles to their use by the sponsor. Only a few participants were aware of platforms that enabled sponsors to view patient counts in the EHR systems of different hospitals/countries, and had piloted, for example, the InSite EHR research platform (Formerly Custodix, Belgium, currently part of TriNetX, USA), but did not continue this use because of the lack of data regarding the Nordic sites. Most participants stated that the use of EHR data by trial sponsors had not increased as expected or desired, mostly because of legislative barriers.

### Characteristics of the Nordic countries in feasibility evaluations

The participants identified some specific features in the Nordic countries when conducting feasibility evaluations.

#### The sites lack the time for clinical research

Some participants found it worrying that investigators did not have sufficient time or interest to conduct clinical trials: even if they were identified by the sponsors, no collaboration emerged.

#### Competitive factors

The participants noted that Nordic sites could and should distinguish themselves from other countries by employing advanced technical solutions and processes for the efficient secondary use of their healthcare data for clinical trials. The Nordic sites were seen to be the most competitive in complex trials with a need for database searches for suitable trial participants, either from disease registries or from EHR data.

#### Future of the EHR query systems

Most participants underlined that the need for patient EHR data in identification of trial participants will be emphasized in the future, especially in trials in rare diseases and in trials on targeted medicines. They perceived that the use of EHR data would expedite obtaining the patient count estimates and to improve the estimates’ accuracy. When requested to describe the ideal future set-up for querying the EHR data, the participants wished to have access to larger entities than single hospitals; even Nordic-wide EHR data lakes with highly secured data protection were emphasized to obtain sufficient coverage of the whole Nordic population, with 27 million inhabitants.^10^

## Discussion

### Site identification and selection

The increased use of data in the feasibility evaluation process seems to have decreased the role that local subsidiaries used to have in country selection. Probably because of that change, the sites’ and investigators’ visibility in databases was found in this study’s observations fundamental for regions or countries to be selected to participate in new clinical trials. In the future, the need to identify certain types of patients more precisely will be important. Low-volume trial countries, such as the Nordic countries, may not get sufficient weight in the site performance databases, but their visibility could be enhanced with advanced technology and by efficient use of patient data, such as EHR data, to meet the needs in the changing landscape of clinical trials. This would also require changes in the legislation, that is, allowing access to the aggregated patient data for third parties. Also, the experience and the local knowledge of the sponsor subsidiaries remain very important for promoting their countries to the global teams.

According to our respondents, site selection appears to be based on trust and relationships built from previous collaborations, but electronic data provide an important support in the selection process. On a local level, the sponsors use electronic data (data on previous site performance) for validating the patient count estimates rather than for identifying the sites. The site selection methods do not seem to be explicitly associated with the success or failure of patient recruitment (Table 2). This investigation should have been performed on the site level (not on the trial level) in order to make more distinct conclusions between the recruitment success and site identification methods.

### Evaluation of access to patients

Over-estimation of the availability of eligible patients is the major reason for the sites’ failure to recruit successfully,^17^ which in turn translates to negative performance data in the databases, reducing the sites’ possibilities to be awarded future trials. According to our interview responses, and reported by others,^18^ quite often the trial sites review their patient potential only after trial initiation, only to realize that they will not be able to fulfil their recruitment goals. Therefore, in the feasibility evaluation, it is crucial that the sites find out the requirements set for them, and that they are enabled to use factual data on their patient counts to adjust the expected number of trial subjects achievable for them. Most sites do understand the importance of formulating the recruitment projections^19,20^ but may not have enough time and resources^21^ or may not have access to sufficient data to perform the required estimations accurately.

According to our respondents, Nordic investigators estimate their capability to recruit trial participants in various ways; some of them use their site’s EHR data for supporting the estimations but most investigators base their estimates only on their previous experience. Earlier studies have reported that the investigators’ most common data source is recruitment data from their own or their colleagues’ previous trials.^19^ The current results indicate that sites providing EHR evidence to sponsors to support their patient count estimates are the most attractive ones.

Getting proper information on the trial is one of the most influential factors for the sites to be able to decide on their participation.^22^ The importance of communication^23^ has not been replaced by the use of data, but various types of data sources have been deployed along with the communication when evaluating the sites’ recruitment projections. Most frequently, sponsors use previous performance data for validating the investigators’ estimates. Because of legislative barriers and the lack of data on Nordic trial sites, the trial sponsors had not yet started to use EHR query platforms, even if they found their use much needed in the future.

In this study, among the trials only run with the sites already known to the sponsor, 5 out of 15 trials failed in their patient recruitment. This strengthens the view that the sites’ patient count estimates are difficult to evaluate^24,25^ in spite of previous collaborations, and that recruitment success is multifactorial.

### Use of EHR data for estimating access to patients

Electronic solutions, such as EHR systems, are regarded as valuable means to increase the accuracy of patient count estimates^8,9^ and thereafter to possibly contribute to recruitment success. In most clinical trials, patients are recruited from the site’s own patient population.^26^ The more accurately investigators are able to estimate how many eligible patients there are at a certain site, the more accurate estimates can be made on the sites’ recruitment target. However, it should be kept in mind that there are always many factors other than access to patients that influence the overall success of recruitment. Examples of these are investigators’ time resources and motivation, and the patients’ willingness to joining a study. Electronic data can be used to support estimating and validating the patient counts, but it can only partly solve the challenges of patient recruitment.

In order to enable efficient, transparent and secure secondary use of EHR data for the purposes of clinical trials, associated legislative aspects must be addressed. The Nordic countries have patient care data in electronic format and all residents are identifiable through unique personal identity numbers. Having the technical capabilities and ability to combine person-based information from multiple sources via a personal identifier is a competitive advantage for the Nordic countries.^10^ This advantage should be exploited for consistent use of EHR data for feasibility evaluations and for increasing the Nordic countries’ visibility in EHR applications, as their use was seen to increase in the future.

## Limitations

Credibility, dependability, transferability^27^ and reflexivity^13^ aspects were considered to evaluate the trustworthiness of the findings. For example, transferability of the results was strengthened by having chosen the four Nordic countries for the study, instead of just one or two. The interview participants represented pharmaceutical companies and contract research organizations over a wide scale and presented trials in various therapeutic areas and in all pre-market phases of clinical drug development, expressing and presenting heterogeneous views on the items discussed. One researcher (N.L.), with a long background in the pharmaceutical industry, conducted all interviews and analyses, which may have affected the reflexivity of the findings. The possible impact of the researcher’s own perceptions was minimized by following a pre-defined interview guide, in a similar manner, with all participants, and by having all interpretations challenged in systematic discussions with A.A. A topic for future research would be the reasons why some sites use EHR data for patient count estimates while others do not, and how they compare in recruitment success. We also wish to highlight that the investigators’ patient count estimates are here described from the sponsors’ point of view. Our respondents’ perceptions may not always be in line with how the investigators see the benefits and challenges in using EHR data for patient count estimates. The investigators’ view would also be a valuable future research topic.

## Conclusion

As the use of various types of data has increased dramatically in the decision-making on trial planning, visibility in the data is crucial for countries and sites to be identified for participation in new clinical trials. It may be difficult for countries with small populations and low volumes of clinical trials to gain visibility and be selected based on previous performance data. They might distinguish themselves from many high-volume countries by developing the use of EHR systems and data lakes for identifying special patient groups with certain characteristics. This can be achieved by enabling EHR legislation and streamlined processes in the hospitals. Overall, site selection appears to be based on trust and relationships built from experience, but electronic data provide the support upon which the trust is based. Estimating the number of potential trial participants is a complex, time-consuming and still largely approximative process for both investigators and trial sponsors. Sponsors seemed to favour sites using EHR data in their patient count estimations because of the promptness and reliability of the estimates. As the sites’ own patients are a common source for actual recruitment of trial participants, it seems that consistent use of the sites’ EHR data already in the feasibility evaluation would have a clear impact on the accuracy of the recruitment estimates and, on its part, would contribute to improved recruitment success.

## Supplemental Material

sj-pdf-1-ctj-10.1177_17407745211038512 – Supplemental material for Clinical trial site identification practices and the use of electronic health records in feasibility evaluations: An interview study in the Nordic countries Click here for additional data file. Supplemental material, sj-pdf-1-ctj-10.1177_17407745211038512 for Clinical trial site identification practices and the use of electronic health records in feasibility evaluations: An interview study in the Nordic countries by Niina Laaksonen, Mia Bengtström, Anna Axelin, Juuso Blomster, Mika Scheinin and Risto Huupponen in Clinical Trials

sj-pdf-2-ctj-10.1177_17407745211038512 – Supplemental material for Clinical trial site identification practices and the use of electronic health records in feasibility evaluations: An interview study in the Nordic countries Click here for additional data file. Supplemental material, sj-pdf-2-ctj-10.1177_17407745211038512 for Clinical trial site identification practices and the use of electronic health records in feasibility evaluations: An interview study in the Nordic countries by Niina Laaksonen, Mia Bengtström, Anna Axelin, Juuso Blomster, Mika Scheinin and Risto Huupponen in Clinical Trials
