# Supplementary material for: Clinical trial site identification practices and the use of electronic health records in feasibility evaluations: An interview study in the Nordic countries
Source: Clin Trials. 2021 Aug 25;18(6):724–31. doi: 10.1177/17407745211038512 (PMC8592101; doi:10.1177/17407745211038512)
Supplement: sj-pdf-1-ctj-10.1177_17407745211038512 – Supplemental material for Clinical trial site identification practices and the use of electronic health records in feasibility evaluations: An interview study in the Nordic countries [file sj-pdf-1-ctj-10.1177_17407745211038512.pdf]

Success of patient recruitment in clinical drug trials in Nordic countries
# - use of electronic data for site identification and patient identification

Thank you for your interest in participating in the interview. It will be done via phone or skype and will contain the following themes. For preparation, please write down your notes in advance. The interview will take max 1 hour.

Please think about 2 of your most important clinical drug trials during 2015-2018 in which you were/are involved and which included one or more Nordic country.

	**Trial 1**	**Trial 2**
1. Which phase (I-IV) and disease was investigated?		
2. Which countries were involved?		
3. How many patients per Nordic country were planned to be included vs how many were actually included?		
4. Is the patient recruitment completed (or ongoing) according to planned schedule?<br>• If Yes, define key success factors for recruiting patients as planned<br>• If No, how much was the recruitment period prolonged from planned? (in months)		
5. Is the trial completed (or ongoing) according to planned schedule?<br>• If Yes, define key success factors for keeping timelines<br>• If No, how much was the trial completion delayed from planned? (in months)		
6. Which were the major factors causing delays in the trial?		
7. Please compare delays caused by patient recruitment to delays caused by other trial-delaying factors?		

Answer options:<br>• Patient recruitment was the major trial-delaying factor<br>• Patient recruitment delays had some effect on trial delay<br>• Patient recruitment was a minor trial-delaying factor<br>• Patient recruitment had no effect on trial delays		
8. Please consider trial activities preceding the recruitment period:<br>Was the recruitment started on schedule or were there delays in preceding start-up activities? Why?<br><br>If start of recruitment period was delayed, was it compensated by efficient recruitment? Yes/No		
9. Have you done protocol feasibility to ensure that the inclusion/exclusion criteria are optimal?		
10. How was the site identification and patient identification done? I.e. From where and how did the study team find out about potential sites and potential patients?<br>Include also comment if Patient organizations were used and whether it was considered useful.		
11. What electronic data (eg. electronic databases, registers, electronic health records or other electronic tools) were used for trial site identification and for patient identification?<br><br>Are there country-specific differences in using electronic data for these purposes?		

<table>
<tr><td>12. Was there benefit from using them? Yes/No
If yes, how much (little/ much/very much) and what kind of benefit?
If No, why?</td><td></td><td></td></tr>
<tr><td colspan="3">These final questions are not restricted to the “2 most important trials” referred to in questions 1.-12. but in general:
What kind of electronic solutions would you regard valuable in future clinical trials when identifying suitable trial sites or trial patients?
In your experience, which types of trials have been most successful in finding suitable patients? Why?</td></tr>
</table>

Operational environment and the role of the respondent, also these will be asked during the interview:

- *What is your job description and history in the company and impact on the site identification and patient recruitment process?*
- *What is your history (in years) in conducting clinical trials?*

All answers will be treated with highest confidentiality. No names of interviewees or their companies will be identifiable from the published results.

Thank you very much for your collaboration!
