# Supplementary material for: Clinical trial site identification practices and the use of electronic health records in feasibility evaluations: An interview study in the Nordic countries
Source: Clin Trials. 2021 Aug 25;18(6):724–31. doi: 10.1177/17407745211038512 (PMC8592101; doi:10.1177/17407745211038512)
Supplement: sj-pdf-2-ctj-10.1177_17407745211038512 – Supplemental material for Clinical trial site identification practices and the use of electronic health records in feasibility evaluations: An interview study in the Nordic countries [file sj-pdf-2-ctj-10.1177_17407745211038512.pdf]

Supplementary material 2. Examples on quotations, codes and categories

Examples on quotations	Code	Sub-category	Category	Main category
"Because it is just not talking about volumes, we are talking about targeted medicine. We are talking about patients with specific mutations. We need to be able to pinpoint where those exact patients are. That is something that is here and will also be more and more in future, I am sure." ID5	The increased need to find specific patients	Increased need for data	Changed needs of data in the feasibility evaluations	**Changing landscape of feasibility evaluations**
"That can be good or bad. Data driven. You can´t argue with data. I mean if you have the correct data, that can be a good tool (for finding sites), but if you don't, you can make some great mistakes." ID14	Risk of incorrect conclusions due to erroneous data	Conclusions from erroneous data	Increased amount of data causing considerations on trust on available data	
"I would say to my colleagues that one should be aggressive (in marketing) and not just accept the thing that we just don't get the trials. It is our obligation as directors in Finland to constantly market (our country), and not just wait whether we get (the trials) or not." ID15	Need for active marketing	Role of Nordic subsidiaries of the pharmaceutical companies	Site identification on global level	**Site identification in two layers**
"No, we don't use any databases because Denmark is a small country. We have had so many studies so we already know them (the sites)." ID20	Databases not used for site identification	Site identification	Site identification in local level	
"And very important is the communication to the potential investigators. Then they know what they are saying "yes" to. That is very important for the feasibility evaluation." ID17	Communication with the investigators	Communication in site identification process		
"It is always when they (trials) are initiated, that is usually the first time when they (investigators) really start to think about the study and where to find the patients.. ..I don't think they take time until studies are initiated." ID1	Investigators estimating patient counts too late	Approximate estimation of patient counts	Investigators evaluating number of potential trial subjects	**Evaluation of sites' access to patients**
"They (the site) were very good in prediction of the study. So we can set up good expectations for Sweden. We did know we have this many patients (at the site)… it is not just a guess, or not even a validated guess… it is a fact that we know, that we have this number of patients." ID9	Trust on investigator estimates because of data evidence	Data evidence provided by the investigators		

"Q: How do you evaluate the investigator's promises to recruit this many patients? A: We take that with caution. They are always more optimistic than the reality is. The physicians are generally very busy; they don't have time to go through all the details in the protocol. That is why we really have to be aware of potential challenges. That is why we perform the feasibility evaluation, anyway. We need to ask very carefully and particularly about those challenges." ID17	Discussions about the challenges	Dialogs with the investigator	Sponsors evaluating the number of potential trial subjects	
"They are requested to do a database search. To make sure they have a database and have looked in the database on how many patients they would recruit." ID14	Request for EHR search	Requesting site's EHR evidence		
"Because you can basically see sites (from the database) that are delivering and not delivering. You are not dependent on what someone else is telling you. You are looking at the data that are in the systems." ID5	Estimating site projections against previous performance data	Investigator databases and previous performance data		
"That said, we also have lot of challenges to ensure data privacy and then also to make the data owners (feel) comfortable how the data are being used. So there are lots of challenges, but also lots of opportunities." ID22	Challenges and opportunities in using EHR data	Use of EHR query tools		
"I don't think some of the hospitals in our region fully understand the competitive environment. I was in South Europe last week, meeting with a few hospitals, and they have done very very well, but they are also very aggressively selling the hospital. We don't do that in the Nordics. Even though we are so much better" ID9.	Competence in clinical trials not marketed	Competence	Competitive factors	**Characteristics of the Nordic countries in feasibility evaluations**
"Well, it is mostly hospital patient record type of data that we need in our trials. It should be structured in a way that enables data searches. And most preferably so that it would be nation-wide (data lake), not one hospital at a time." ID7	Searchable patient data in big data lakes needed	Need for EHR data	Future of the EHR query systems	
